# The Molecular Epidemiology and Transmission Dynamics of HIV Type 1 in a General Population Cohort in Uganda

**DOI:** 10.3390/v12111283

**Published:** 2020-11-10

**Authors:** Deogratius Ssemwanga, Nicholas Bbosa, Rebecca N. Nsubuga, Alfred Ssekagiri, Anne Kapaata, Maria Nannyonjo, Faridah Nassolo, Alex Karabarinde, Joseph Mugisha, Janet Seeley, Gonzalo Yebra, Andrew Leigh Brown, Pontiano Kaleebu

**Affiliations:** 1 Medical Research Council (MRC)/Uganda Virus Research Institute (UVRI) and London School of Hygiene and Tropical Medicine (LSHTM) Uganda Research Unit, Entebbe 256, Uganda; Nicholas.Bbosa@mrcuganda.org (N.B.); nrebeccansubuga@gmail.com (R.N.N.); Anne.Kapaata@mrcuganda.org (A.K.); Maria.Nannyonjo@mrcuganda.org (M.N.); Faridah.Nassolo@mrcuganda.org (F.N.); Alex.Karabarinde@mrcuganda.org (A.K.); Joseph.Mugisha@mrcuganda.org (J.M.); Janet.Seeley@mrcuganda.org (J.S.); Pontiano.Kaleebu@mrcuganda.org (P.K.); 2 Department of General Virology, Uganda Virus Research Institute, Entebbe 256, Uganda; assekagiri@gmail.com; 3 Department of Global Health and Development, London School of Hygiene and Tropical Medicine, 15-17 Tavistock Place, London WC1H 9SH, UK; 4 The Roslin Institute, Royal (Dick) School of Veterinary Medicine, University of Edinburgh, Easter Bush Campus, Edinburgh EH25 9RG, UK; Gonzalo.Yebra@ed.ac.uk; 5 Institute of Evolutionary Biology, University of Edinburgh, Edinburgh EH9 3FL, UK; A.Leigh-Brown@ed.ac.uk

**Keywords:** molecular epidemiology, HIV-1, transmission networks, phylogenetic, demographic, general population, subtype, bayesian, phylogeography

## Abstract

The General Population Cohort (GPC) in south-western Uganda has a low HIV-1 incidence rate (<1%). However, new infections continue to emerge. In this research, 3796 HIV-1 *pol* sequences (GPC: *n* = 1418, non-GPC sites: *n* = 1223, Central Uganda: *n* = 1010 and Eastern Uganda: *n* = 145) generated between 2003–2015 were analysed using phylogenetic methods with demographic data to understand HIV-1 transmission in this cohort and inform the epidemic response. HIV-1 subtype A1 was the most prevalent strain in the GPC area (GPC and non-GPC sites) (39.8%), central (45.9%) and eastern (52.4%) Uganda. However, in the GPC alone, subtype D was the predominant subtype (39.1%). Of the 524 transmission clusters identified by Cluster Picker, all large clusters (≥5 individuals, *n* = 8) involved individuals from the GPC. In a multivariate analysis, clustering was strongly associated with being female (adjusted Odds Ratio, aOR = 1.28; 95% CI, 1.06–1.54), being >25 years (aOR = 1.52; 95% CI, 1.16–2.0) and being a resident in the GPC (aOR = 6.90; 95% CI, 5.22–9.21). Phylogeographic analysis showed significant viral dissemination (Bayes Factor test, BF > 3) from the GPC without significant viral introductions (BF < 3) into the GPC. The findings suggest localized HIV-1 transmission in the GPC. Intensifying geographically focused combination interventions in the GPC would contribute towards controlling HIV-1 infections.

## 1. Introduction

Since the beginning of the HIV epidemic in the early 1980s, several African countries including Uganda have registered significant progress in controlling the epidemic [1]. In Uganda, HIV-1 incidence was shown to reduce in the 1990s [2]. This success was mainly due to the Government’s early acknowledgement of the impact of the epidemic and commitment to scale down the epidemic through a multifaceted approach to prevention and care [3]. Findings from the 2011 Uganda AIDS Indicator Survey (UAIS) showed an overall HIV-1 prevalence of 7.3% among adults aged 15–49 years with a higher prevalence among women (8.3%) than among men (6.1%) [4]. This survey further showed a higher prevalence of HIV among women in urban areas (10.7%) compared to rural areas (7.7%); however, HIV prevalence was the same for men in urban and rural areas (6.1%). The most recent 2016 Uganda Population-Based HIV Impact Assessment (UPHIA) survey indicated a fall in the national HIV-1 prevalence to 6% compared to 7.3% in 2011 [5]. The report further showed that prevalence among women and men declined from 8.3% and 6.1% in 2011 to 7.5% and 4.3% in 2016 respectively. In urban areas, prevalence declined from 8.7% to 7.1% while in rural areas it fell from 7.0% to 5.5%.

However, recent reports have shown extremely high prevalence and incidence among known key populations such as fisherfolk, sex workers, truck drivers, the armed forces, and individuals involved in the public transport sector among others [6]. In the fishing communities of Lake Victoria, incidence and prevalence have been reported at 6 per 100 person years at risk (PYAR) [7] and 28.8% [8], respectively. A high proportion of this population reported high-risk sexual behaviours including multiple sex partners, frequent change of partners, low condom use even with partners known to be living with HIV, transactional sex and sex under the influence of alcohol or drugs [9,10,11]. Studies conducted among female sex workers in Uganda have shown an incidence of about 3/100 person years [12,13] and prevalence of 37% [14].

Over the years, several research groups have been involved in research aimed at understanding factors associated with HIV-1 transmission, pathogenesis, prevention and vaccine development in Uganda. The Medical Research Council/Uganda Virus Research Institute and London School of Hygiene & Tropical Medicine Uganda Research Unit (MRC/UVRI & LSHTM) established the General Population Cohort (GPC) in Kalungu District (formerly a part of Masaka District), south-western Uganda to examine the trends in HIV-1 prevalence and incidence, their determinants and pathogenicity [15]. Data collected between 1990–2010, showed that the HIV-1 incidence in the GPC was generally fluctuating below 1 per 100 PYAR [16], although new HIV-1 infections continued to emerge in this population [2,17]. We hypothesize that this population is not highly mobile as most residents depend on small scale agriculture, and it remains unclear if these new cases are attributed to migration, transmission from within or without the population. In this study, we use phylogenetics and study participant demographic data to understand the HIV transmission dynamics in this population to inform prevention.

## 2. Materials and Methods

### 2.1. Study Setting, Participants and Sample Collection

The GPC, enrolled approximately 10,000 adults in 1989/1990 from a cluster of 15 villages to study the epidemiology of HIV-1 infection [18]. In 1990, a random selection of one-third of seropositive adults identified in the initial GPC serosurvey round were invited to enrol into a natural history cohort (NHC) to study the disease progression of HIV-1-infected participants within the GPC [19]. In 1999, an additional 10 villages with a population of about 8000 were added to the GPC to improve the precision of HIV prevalence and incidence estimates [2,7]. Following the introduction of free antiretroviral therapy (ART) in Uganda in 2004, the NHC, was renamed the rural clinical cohort (RCC) [20]. Once enrolled into the RCC, study participants attend the clinic every three months for clinical history, examination and blood sampling. In the RCC, HIV-1 infected participants are encouraged to bring their partner(s) for voluntary counselling and testing (VCT) and possible enrolment.

Over the last 30 years, the GPC has provided information on the prevalence and incidence of HIV-1 infection [19,21], how sexual behaviour contributed to HIV-1 acquisition and factors strongly associated with increased risk of HIV infection [22]. Subsequent annual serosurveys of the GPC have identified new HIV-seropositive participants, who have been recruited as incident cases, the majority of whom have estimated dates of seroconversion. All HIV patients enrolled in the GPC receive ART services/care at the MRC/UVRI & LSHTM clinic sited at the main town in the GPC area.

We additionally recruited study participants living with HIV who were not enrolled in the GPC but were found within the larger GPC catchment area. This was done to increase our sampling coverage and minimise missing out other eligible HIV positives identified within the geographical confines of the GPC. This group termed “non-GPC” composed of individuals who either accessed HIV care services at the MRC/UVRI & LSHTM clinic despite not being enrolled in the GPC or at other health facilities in the GPC area. In the non-GPC, convenience sampling was done for all HIV-1 positive individuals who consented to participate in the study and were at least 16 years old.

In the study presented here, we analysed a total of 3796 HIV-1 pol sequences from samples collected between 2003–2015 from the GPC (*n* = 1418), non-GPC (*n* = 1223) and from other sites in Central (*n* = 1010) and Eastern (*n* = 145) Uganda. As described previously [23,24], we used a biometric fingerprint-scanning device with all study participants to avoid duplicate enrolments and also increase our certainty of the clusters identified in the HIV-1 transmission network analyses.

### 2.2. Laboratory Methods

#### DNA Extraction, PCR Amplification and DNA Sequencing

Two different approaches were used to extract and amplify HIV-1 nucleic acids from samples. For samples of patients who were not on ART or had detectable viral loads, viral RNA was extracted from plasma (140 µL) using the QIAmp Viral RNA mini kit (Qiagen Inc., Valencia, CA, USA) as previously described [25]. In this group, the entire protease (codons 1–99) and amino terminus of reverse transcriptase (codons 1–320) were amplified using a one-step RT-PCR kit (Qiagen, Valencia, CA, USA) and sequenced using an in-house protocol as described elsewhere [20,25,26,27]. Sequencing was done using the ABI 3500 Genetic Analyzer (Applied Biosystems, Foster City, CA, USA) and sequence contigs assembled using both Sequencher v5.2.4 (Gene Codes Corporation, Ann Arbor, MI, USA) and RECall Software [28] for quality control purposes. Basic phylogenies using Maximum Likelihood (ML) trees [29] were performed to determine sequence relatedness and to rule out contaminations. For low-viremic samples like in the case of patients on ART, pro-viral DNA was extracted from cell pellets. Here, whole blood was processed to obtain the pellets by centrifuging at 3000 g for 10 min and the provirus was extracted using the QIAamp Viral DNA kit (Qiagen, Hilden, Germany). Nested PCR was performed to amplify the HIV-1 *pol* (protease codon 1–99 and the amino terminus of reverse transcriptase codons 1–320) using gene specific primers described elsewhere [25]. The sequencing and downstream analysis was the same as that done for the viral RNA approach described above. We included participants on ART to increase our sampling coverage and to encompass all possible transmission sources.

### 2.3. HIV Subtyping

We performed viral subtyping of the generated nucleotide sequences using COMET [30] and REGA [31] software. Sequences that were unassigned by both software were considered unknown.

### 2.4. Phylogenetic and Transmission Network Analysis

Phylogenetic analysis was used to identify HIV-1 transmission networks. Subtype reference sequences of HIV-1 group M from the Los Alamos HIV Sequence database [32], were used to automatically align the generated sequences using ClustalX version 2.0 [33]. The software ViroBLAST was used to scan public databases for sequences similar (genetic similarity of ≥95%) to our query sequences and for checking contamination [34]. We further used the ElimDupes software [35] to compare the ViroBLAST generated, subtype references and the study sequences to eliminate any duplicate sequences. The sequences obtained using ViroBLAST were used with the subtype reference sequences to construct ML trees using PhyML Software [36], and the reliability of tree topologies was estimated by bootstrap analysis (1000 replicates) [37]. Phylogenetic HIV-1 transmission pairs and clusters (containing ≥ 2 sequences) were identified at a maximum genetic distance of 1.5% using the Cluster Picker software [38]. In this study, pairs (*n* = 2) and clusters (*n* > 2) are all referred to as clusters. We used the Cluster Picker software to identify HIV-1 transmission networks on reconstructed ML trees because this method provides clade support (bootstrap or posterior probability) for linked sequences on a phylogenetic tree in addition to using the maximum pairwise genetic distance within the cluster. Pairwise genetic distances are calculated for all sequences in a given dataset and if the highest determined genetic distance of the bunch is less than or equal to the set maximum genetic distance threshold, then the group of sequences is identified as a cluster. Alternatively, if the maximum pairwise distance is greater than the set threshold, then the cluster is excluded [38]. Additionally, the clusters identified in this way are similar to those defined by time-resolved phylogenies [38]. In contrast, other commonly used programs like HIV-TRACE [39] do not infer a phylogenetic tree but rely on genetic linkage and employ a single-linkage algorithm, where a sequence is determined as linked to another if its genetic distance to only one other in a cluster is below a predefined threshold [38,39]. In the study presented here, the use of a genetic distance threshold of 1.5% was based on our previous analyses in which evaluations were done across a range of different genetic distance thresholds [23,40]. A stringent genetic distance cut-off of between 0.01 and 0.02 was considered ideal in our study setting to identify potential transmission networks while minimizing the likelihood of finding false associations [40]. 

### 2.5. Bayesian Phylogeographic Analysis

We performed a Bayesian phylogeographic analysis in BEAST v1.8.4 on pure HIV-1 subtype A and D sequences (excluding HIV recombinant forms and those that could not be subtyped by COMET or REGA herein referred to as unknown) that formed clusters to reconstruct the spatial dynamics of viral diffusion between the GPC, non-GPC and other sampled locations as previously described [23]. An asymmetric discrete traits substitution model with a Bayesian Stochastic Search Variable Selection (BSSVS) method was used to estimate transition rates between locations [23,41,42]. First, we examined the temporal signal of HIV-1 sequences using TempEst v1.5 [43] and implemented a Bayesian Markov Chain Monte Carlo (MCMC) method in BEAST v1.8.4 for 300 million generations sampling after every 20,000th iteration. To obtain the optimum combination of model parameters for the BEAST runs, we used the path sampling/stepping-stone method [44] to compare marginal likelihood estimates of different substitution models (SRD06 [45] and Yang 96 [46]), demographic models (Bayesian Skygrid [47,48] and GMRF Skyride [49]) and molecular clocks (strict and relaxed [50,51,52]). We used an uncorrelated lognormal relaxed molecular clock with the SRD06 model of nucleotide substitution and a coalescent Skygrid tree prior for subtype D HIV-1 *pol* sequences and a Yang 96 model of nucleotide substitution and Skyride GMRF demographic model for the subtype A sequence dataset. An value of 1.5 × 10^−3^ substitutions/site/year was based on estimates from a previous studies [23,41]. Historical sequences that were sampled in the 1980s during the early years of the HIV epidemic in Uganda were included in the sequence datasets to improve the temporal signal for the BEAST analysis and the convergence of the MCMC runs [41]. Convergence of the MCMC results was examined in TRACER [53] based on the effective sample size (ESS) of >200 after a 10% burn-in. Maximum Clade Credibility (MCC) trees that represented a summary of the posterior tree distributions were generated with TreeAnnotator [54] and visualized in FigTree [55]. We reconstructed the viral diffusion patterns between locations in SPREAD [56] and examined the viral migration profiles within and outside the GPC. A Bayes Factor (BF) test was applied to determine significant non-zero transition rates and a cut-off of BF  =  3 was used [41,56,57].

### 2.6. Quality Control and Sequence Accession Numbers

For quality control purposes, samples from the identified clusters underwent in-house Quality Assurance checks to rule out cross-contamination. To avoid breaching patient confidentiality and possible deductive disclosure of study participants in the GPC where at least >95% of all inhabitants are surveyed, we submitted a random sample of 10% of HIV-1 sequences to Genbank [58,59] (under accession numbers MT992962–MT993341).

### 2.7. Statistical Analysis

Demographic and clinical data were summarized using absolute numbers and relative proportions for categorical variables. This included information obtained from questionnaires (place of residence, marital status, age, gender, ART status). Particularly in the GPC, demographic and clinical data were drawn mostly from the GPC census and clinic records while for non-GPC sites where convenience sampling was done, short questionnaires were administered. Median and inter-quartile ranges were used to summarize continuous variables. Comparisons of categorical demographic factors and cluster membership were based on chi-square tests of independency. Associations of the probability of belonging to a transmission cluster with various demographic characteristics (gender, age, subtype, location and ART) were investigated using logistic regression. All analysis was performed in R software version 3.6.2.

### 2.8. Ethical Considerations

Ethical clearance was obtained from the Uganda Virus Research Institute (UVRI) Research and Ethics Committee (REC) on 24/06/13 (Federal Wide Assurance (FWA) No. 00001354, Project identification code: GC/127/13/06/27) and the Uganda National Council for Science and Technology (UNCST) on the 12/09/13 (FWA No. 00001293, Project identification code: HS 1432). This study was nested as part of the MRC/UVRI & LSHTM HIV-1 Molecular Epidemiology study which aimed to determine the trends of HIV-1 subtypes and transmission linkages among high-risk groups and the general population in Uganda. All participants were recruited voluntarily and provided written informed consent.

## 3. Results

### 3.1. Summary Characteristics of Study Populations

A total of 3796 enrolled participants with sequence, demographic and clinical data were analyzed (Table 1). The median age of participants was 34 years [inter-quartile range (IQR) of 26–45 years]. This composed of 1733 (45.7%) male and 2063 (54.3%) female participants. A total of 1418 (37.4%) participants were from GPC, 1223 (32.2%) from the non-GPC, 1010 (26.6%) from the Central region and 145 (3.8%) from the Eastern region. The majority of study participants were on ART (74.5%) and 970 (25.5%) were ART naïve. The overall subtype distribution was A1 (41.1%), D (34.3%), recombinants (10.9%), unknown (1.7%), C (11.9%) and G (0.1%).

### 3.2. HIV-1 Subtyping

HIV-1 subtype A1 was the most prevalent strain in the GPC catchment area (GPC and non-GPC sites) (39.8%), central (45.9%) and eastern (52.4%) Uganda. However, in the GPC alone, subtype D was the predominant subtype (39.1%) (Table 2).

### 3.3. Transmission Networks

#### 3.3.1. Cluster Size Distribution 

Of the 3796 participant sequences included in the analysis, we identified a total of 524 transmission clusters (cluster size of 2–7) that included 1198 sequences (31.6%) using Cluster Picker. Of these clusters, 409 (78.1%) composed of two individuals, 94 (17.9%) composed of three individuals, 13 (2.5%) composed of four individuals, 4 (0.8%) composed of five individuals, 2 (0.4%) composed of six individuals and 2 (0.4%) composed of seven individuals (Table 3, see also supplementary Table S1). The distribution of HIV-1 subtypes across all clustered sequences (*n* = 1198) was as follows: subtype A (452, 37.7%), subtype D (396, 33.1%), subtype C (214, 17.9%), recombinants and other unknown HIV-1 variants (136, 11.4%).

#### 3.3.2. Characteristics of Transmission Clusters

A majority (187, 35.7%) of all clusters (*n* = 524) were from the GPC, followed by those from central Uganda (177, 33.8%), non-GPC sites (148, 28.2%) and Eastern Uganda (12, 2.3%). Similarly, the majority of individuals in the larger clusters (≥5 individuals) were from the GPC. Two large clusters (C93, cluster size = 7 and C448, cluster size = 5) composed of pure subtypes A1 and D sequences included individuals from only the GPC. One large cluster (C290) contained subtype D sequences from all four locations. All clusters of size ≥5 contained pure subtype (A1 and D) sequences with the exception of cluster C272 that contained inter-subtype recombinants (ISR) (Table 4).

Among the 1198 persons in the 524 clusters, the assortativity coefficient *r* (*r* = 1 indicates perfect assortativity) was 0.47 for study location, 0.09 for age and −0.31 for gender indicating non-assortative mixing for gender but assortative mixing for location and age. Assortativity differed by cluster size for location (clusters of size 6 were non-assortative) and age (clusters of size 5 and 7 were non-assortative). There was non-assortative mixing of gender at all cluster sizes.

#### 3.3.3. Factors Associated with Transmission Cluster Membership

A chi-square test of independency that considered demographic/clinical details showed significant association for cluster membership for location, being on ART and HIV subtypes with the exception of gender and age (Table 5). Table 5 presents the results of a multivariate analysis showing the association between cluster memberships and certain demographic factors. Individuals on ART were less likely to belong to a cluster than those that were ART naïve (adjusted Odds Ratio, aOR = 0.025; 95% CI, 0.020–0.032; *p* < 0.001). Women were also more likely to belong to a cluster than men (aOR = 1.28; 95% CI, 1.06–1.54; *p* = 0. 0102). Taking location into account, individuals from the GPC (aOR = 6.90; 95% CI, 5.22–9.21, *p* < 0.001) and Non-GPC (aOR = 5.12; 95% CI, 3.86–6.85; *p* < 0.001) were more likely to cluster than those from central Uganda. Sequences with inter-subtype recombinants were significantly less likely to cluster than subtype A1 (aOR = 0.68; 95% CI, 0.49–0.92; *p* = 0.0139). Considering different age groups, individuals older than 25 years were significantly more likely to belong to a cluster. Study participants aged 45–54 years were two times more likely to belong to a cluster (aOR = 2.22; 95% CI, 1.61–3.08; *p* < 0.001) than those between 15–25 years.

### 3.4. Phylogeographic Analysis

**Figure 1:**
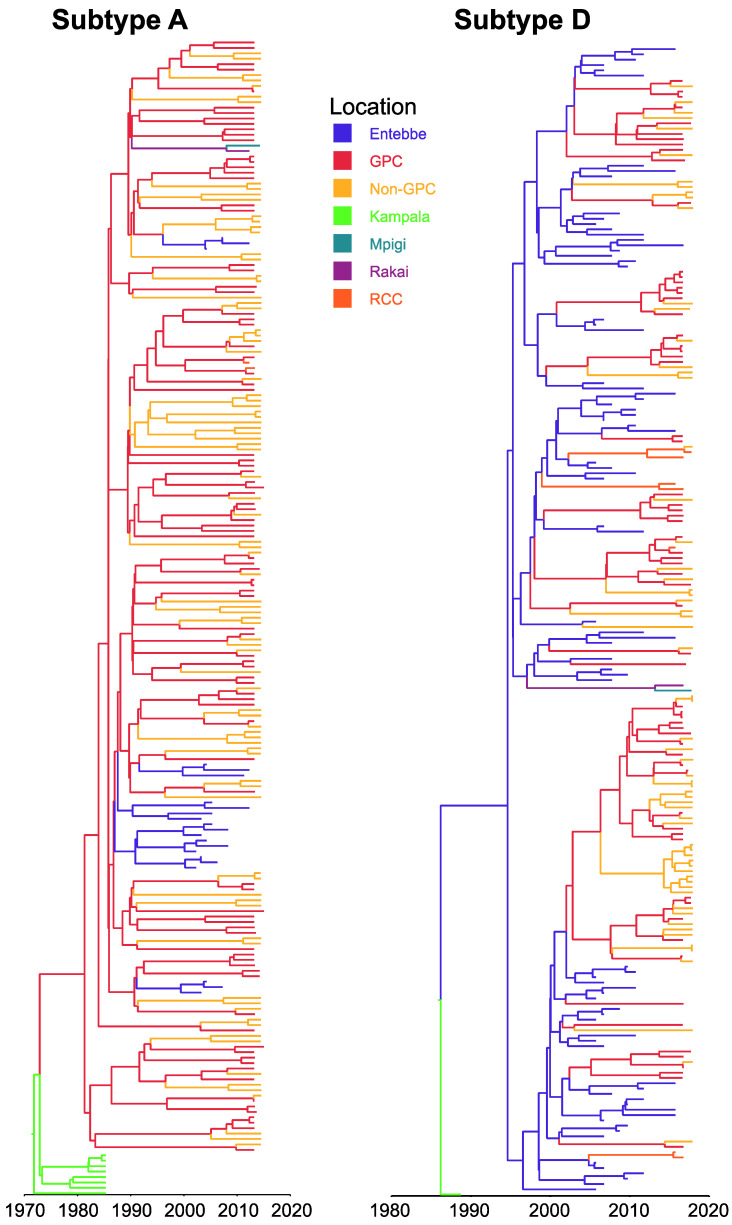
Location-annotated Maximum Clade Credibility (MCC) trees. (**Subtype A**) MCC tree reconstructed from HIV-1 *pol* subtype A sequences from the GPC, areas outside but neighbouring the GPC (non-GPC), historical sequences obtained during 1986 (Kampala) and from other sites (Entebbe, Mpigi, Rakai, rural clinical cohort; RCC). (**Subtype D**) MCC tree reconstructed from HIV-1 *pol* subtype D sequences. The corresponding estimated times to the most recent common ancestor (TMRCA) were 1972 (1961–1975) and 1985 (1979–1987) for subtype A and D respectively. The branches on the tree are coloured according to the location and the time scale at the bottom is in calendar years.

**Figure 2:**
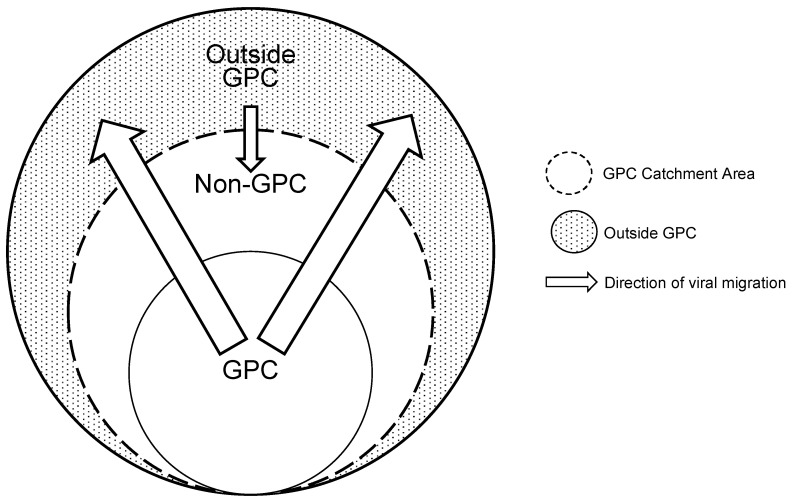
Schematic showing the viral diffusion in the GPC and other external locations. The patterns of virus flow in these communities suggests significant dissemination (BF > 3) of viral lineages mostly outwards and away from the GPC into other areas located outside the GPC with no substantial viral migration (BF < 3) into the GPC. The thicker arrows represent very strong support for viral dissemination (BF > 100) from the GPC while the smaller arrow indicates viral introductions from areas outside the GPC. The size of the arrows is proportional to the level of support for viral diffusion with the thicker arrows indicating stronger support.

We analysed a subsample of 429 HIV-1 *pol* sequences (217 subtype A (GPC = 66, non-GPC = 64, other sites including historical sequences = 87) and 212 subtype D (GPC = 92, non-GPC = 87, other sites including historical sequences = 33) that formed viral transmission clusters to assess the viral diffusion patterns [23,60] in the GPC and other locations. We observed well supported viral migration (BF > 3) in the GPC area without substantial viral dissemination (BF < 3) into the GPC. In both subtype A and D sequence datasets, viral migration from GPC to non-GPC was very strongly supported (BF > 100) [61]. To a lesser extent, we observed some viral introductions into the GPC area from Mpigi (~90 km North East of GPC) and Entebbe (~115 km North East of GPC) (BF > 3), locations that are interlinked by major highways. Overall, viral diffusion tended to flow outwards and away from the GPC. Figure 1 shows a location-annotated Maximum Clade Credibility (MCC) tree for phylogeographic inferences while Figure 2 is a schematic representation of the viral migration patterns between locations.

## 4. Discussion

In this study, we performed a phylogenetic and demographic characterization of the genetic diversity and transmission dynamics of HIV-1 in the GPC located in southwest Uganda. We found subtype D to be the most prevalent subtype in the GPC while subtype A was the predominant strain in sequences sampled from the larger GPC catchment area (GPC and non-GPC), central and southern Uganda. In Uganda, the distribution of HIV-1 subtypes is generally geographically defined based on genotyping of partial viral genes like *pol*, *gag* or *env*, with subtype A more common in the central and northern region [62,63] and subtype D in the southern regions of the country [20,64,65]. In our previous study in the GPC, the proportions of subtype D and subtype A HIV-1 variants was estimated at about 45% and 28%, respectively, based on *gag*/*env* sequences [20]. In the same study, a nonsignificant decrease in the proportion of subtype D strains and an increase in the proportion of subtype A viruses was observed. In our present study based on HIV-1 *pol*, the proportions of subtype D (39.1%) and subtype A (35.3%) viruses in the GPC were comparable, which could suggest an increase in the proportions of subtype A strains in the cohort. Furthermore, the dominance of HIV-1 subtype A in the larger GPC catchment area that includes both GPC and non-GPC sites, suggests that that viral flows do occur in and out of this area.

In the transmission network analysis, all the large clusters (≥5 individuals, *n* = 8) were composed of individuals from the GPC (Table 4) with some of the clusters consisting entirely of linked sequences from the GPC. In our previous study [20] using phylogenetic analysis and participant partnership histories to identify transmission networks, we observed several transmission clusters that were suggestive of high-risk sexual behaviour in the GPC. Consistent with the results presented here, we noted that majority of phylogenetic linkages were within the GPC although clusters involving individuals from other groups were also found. Furthermore, HIV-1 transmission was more likely to occur in individuals who were resident in the GPC (aOR = 6.90; 95% CI, 5.22–9.21, *p* < 0.001) than residents from other study sites (see Table 5). This would suggest that the HIV-1 transmission patterns in the GPC are to a large extent more localized. Potential viral transmission in this study was associated with being ART naïve, being female and being above 25 years of age. The observation that the risk of HIV transmission was associated with older individuals >25 years is consistent with findings from our recent study [24]. An estimated 20.5% (84/409) of all pairs identified consisted of female-female pairs suggesting a larger proportion of unreached men in inferred HIV transmission networks that are still problematic in HIV intervention efforts and present challenges in molecular epidemiological analyses. We also observed clustering between individuals on ART and those that were ART naïve. On a phylogenetic tree, monophyletic clades of HIV sequences from ART and ART naïve persons are represented by longer and shorter branch lengths, respectively, indicating the possibility of transmitting virus by patients on ART. Suboptimal adherence to treatment has been identified as one of the major causes of not achieving viral suppression and increasing the likelihood of HIV transmission in ART experienced patients [66]. Although clustering was most likely associated with individuals who were not on ART, it is important to consider the implications of clusters identified between ART and ART naïve individuals in terms of adherence to treatment and interventions in the GPC. Overall, more clusters containing subtype A sequences where identified in viral transmission networks compared to subtype D with the exception of the larger clusters (≥5 individuals) where subtype D was more common due to the majority of linked individuals originating from the GPC. This implied that subtype A sequences were generally more likely to cluster than subtype D in agreement with our previous findings showing clustering with subtype D to be less likely [40]. However, in this study, inter-subtype recombinant sequences were less likely to cluster (adjusted Odds Ratio, aOR = 0.51, 95% CI 0.49–0.92) in contrast to a study by Kiwuwa-Muyingo et al. [40] that was conducted in a high-risk fisherfolk population group. This difference in results with respect to recombinants is likely to be due to the sequences analysed in our study being drawn from a relatively lower-risk population where recombination is expected to be less common than in key populations.

We sought to investigate the spatial viral diffusion pattern in the GPC and other neighbouring or remote locations using a Bayesian phylogeographic analysis. Viral dissemination out of the GPC was significant without any substantial viral introductions into the GPC suggesting that emerging infections from this low-incidence cohort are mostly from within the GPC. Furthermore, the outward viral diffusion pattern could suggest that travel by residents living in rural GPC communities to the more urban neighbouring areas for trade or work-related activities is not uncommon. The major economic activity of GPC residents is mostly small-scale agriculture (68%) [7] and as such, in-migration is likely to be more stable relative to out-migration as shown in a previous study done in the neighbouring Rakai district [67]. This also implies that partner exchange rates within the rural communities may be lower relative to those in the neighbouring urban areas. The heterogeneity of the HIV-1 epidemic in the GPC (situated about 16 km from the trans-African highway) and neighbouring high-risk populations like the fishing communities (approximately 40 km apart) has previously been described [7] and the viral migration patterns inferred in this study fit well with a diffusion model that supports the flow of virus from this low risk cohort to other higher-risk populations [23,24,68]. Nonetheless, the term ‘lower-risk population’ is a subjective over-simplification of the dynamics of HIV-1 transmission in the GPC as shown by the presence of large HIV-1 clusters and the inferred viral migration patterns. This could also explain the fluctuating HIV-1 incidence trends that have been observed over the years in this cohort.

This study has some limitations. First, subtyping of HIV-1 variants was based on consensus sequences of the *pol* gene. However, with the advent of cheaper next generation sequencing technologies, more near full-length viral genomes have become available and genotyping based on deep sequences has shown that intersubtype A1/D recombinant viruses are highly prevalent (~40%) in southwest Uganda [69,70]. Furthermore, HIV-1 near full length genomes provide better sensitivity in identifying clusters in the inferred viral transmission networks. Secondly, the GPC was sampled more intensely relative to the non-GPC locations where convenience sampling was more commonly done. In the GPC, more comprehensive sampling was implemented that included at least 90% of those enrolled in the cohort through the annual house-to-house demographic and serological surveys. In contrast, we only included those individuals who attended care at the time of recruitment or those on scheduled visits at the ART clinics for the non-GPC sites, which likely comprised a sampling bias. The suboptimal sampling in the non-GPC was as a result of missing those individuals who were living with HIV-1 but were not linked to care. Persons living with HIV but not engaging in care are a major challenge that needs to be addressed in HIV testing/treatment programs [71,72] as well as in molecular epidemiological analyses [24]. Nonetheless, including non-GPC samples in our study provided additional insights into the HIV-1 transmission dynamics beyond the GPC. The more intense sampling in the GPC could also explain the larger number of HIV-1 clusters identified in the GPC (35.7%) relative to those detected from the central area (33.8%) and other non-GPC sites (28.2%), although more pairs than clusters would be expected from the ‘low-risk’ cohort. The observed cluster density (number of clusters divided by the number of sequences) was approximately 18%, 13% and 12% in the central area, GPC and non-GPC, respectively, suggesting a non-significant difference between the cluster density in the GPC and neighbouring non-GPC with the exception of the central region where the cluster density differed significantly from both locations. It would be expected that the lower sampling proportion in the larger central region would give rise to a downward bias in the number of clusters observed. However, because a relatively larger number of clusters was detected there indicates that populations that are at higher risk of HIV-1 transmission were sampled from the central area relative to the GPC and non-GPC. This would imply that the contact networks among which HIV-1 transmission occurs reflect a smaller fraction of the population. Therefore, even with a recruitment bias that resulted from less comprehensive convenience sampling from the clinics and other non-GPC sites, comparable inferences were made in this study. Thirdly, in the phylogeographic analysis, we observed a more recent TMRCA for subtype D compared to an earlier study [41] which could be explained by our sequence selection where we had an overall fewer subtype D and more subtype A sequences from the 1980s a period when subtype D was the majority subtype. Furthermore, to minimize biases related to the ancestral state reconstruction and estimations of the TMRCA [73], HIV-1 recombinant sequences and those classified as unknown were excluded from the analysis in the BEAST program. We however down sampled HIV-1 sequences from overrepresented locations or dates to analyse a representative subsample of sequences as described under methods for comparable results. Additionally, the HIV-1 sequences included in our study spanned a period of over 10 years and this broader temporal range allowed for a better calibration of our molecular clock and phylogeographic inference estimations. Lastly, we did not test for the contribution of factors that are predictive of the observed spatial diffusion process using other phylogeographic parameters like the generalized linear model that could incorporate geographical distance to assess the interaction between places or other economic, agricultural or environmental causes [74,75]. However, this will be a consideration for our future analyses.

## 5. Conclusions

Findings from this study suggest that viral dissemination from the GPC to areas beyond the cohort occurs more frequently and human migration dynamics may play a key role. Studies done in Rakai district (southwestern Uganda) showed that migration influenced risk of HIV [67,76]. Additionally, because HIV-1 transmission was strongly associated with being resident in the GPC with a majority of individuals in the larger clusters coming from this cohort, this could imply that HIV-1 transmission in the GPC is largely localized and that emerging infections are likely from within the cohort. High-risk sexual behaviour involving older individuals (>25 years) living with HIV-1 may drive recurring new infections in this cohort. Therefore, geographically focused combination interventions in the GPC would have the benefit of controlling infections in the GPC and surrounding communities but should incorporate strategies that foster increased testing and linkage to care, adherence to ART and address high-risk sexual behaviour to limit larger transmission chains. This study further highlights the importance of complementing epidemiological investigations with molecular epidemiological-based phylogenetic studies in informing interventions that are aimed at controlling the HIV epidemic.

## Figures and Tables

**Table 1:** Demographic and Clinical Characteristics of Study Population.

Characteristic	Non-Clustered *n* = 2598 *N* (%)	Clustered *n* = 1198 *N* (%)	Overall *n* = 3796 N (%)	*p*-Value
**Study Location**				<0.001
Central	851 (32.8)	159 (13.3)	1010 (26.6)	
East	125 (4.8)	20 (1.7)	145 (3.8)	
GPC	848 (32.6)	570 (47.5)	1418 (37.4)	
Non-GPC	774 (29.8)	449 (37.5)	1223 (32.2)	
**Sex**				0.8101
Male	1190 (54.1)	655 (54.7)	1733 (45.7)	
Female	1408 (45.9)	543 (45.3)	2063 (54.3)	
**ART**				<0.001
Naïve	178 (6.9)	791 (66.0)	970 (25.5)	
On ART	2419 (93.1)	406 (34.0)	2825 (74.5)	
Unknown	1 (0.04)	1 (0.1)	2 (0.1)	
**Age**				0.9847
15–24	589 (22.7)	271 (22.6)	860 (22.7)	
25–34	728 (28.0)	339 (28.2)	1067 (28.1)	
35–44	747 (28.8)	335 (28.0)	1082 (28.5)	
45–54	308 (11.9)	148 (12.4)	456 (12.0)	
>54	226 (8.7)	104 (8.7)	330 (8.7)	
**Subtype**				<0.001
A1	1138 (43.8)	452 (37.7)	1590 (41.9)	
C	187 (7.2)	214 (17.9)	401 (10.6)	
D	917 (35.3)	396 (33.1)	1313 (34.6)	
G	4 (0.2)	0 (0.0)	4 (0.1)	
Inter-subtype recombinants and other unknown HIV variants	352 (13.5)	136 (11.4)	488 (12.9)	

GPC, general population cohort; ART, antiretroviral therapy.

**Table 2:** Distribution of HIV pol sequences according to location.

Location				
HIV Variants	GPC Catchment Area (GPC/non-GPC)	Central	East	Total
A1	1050 (39.8%)	464 (45.9%)	76 (52.4%)	1590
D	907 (34.3%)	368 (36.4%)	38 (26.2%)	1313
C	349 (13.2%)	47 (4.7%)	5 (3.4%)	401
G	2 (0.1%)	2 (0.2%)	--	4
Inter-subtype Recombinants/unknown	333 (12.6%)	129 (12.8%)	26 (17.9%)	488
All	2641	1010	145	3796

GPC, general population cohort.

**Table 3:** Cluster size distribution.

Cluster Size	No. of Clusters	Proportion (%)	Gender			
			F-F (%)	M-F (%)	M-M (%)	M:F Ratio
2	409	78.1	84 (20.5)	287 (70.2)	38 (9.3)	362:455
3	94	17.9	3 (3.2)	90 (95.7)	1 (1.1)	137:145
4	13	2.5	--	13 (100)	--	22:30
5	4	0.8	--	4 (100)	--	8:12
6	2	0.4	--	2 (100)	--	8:4
7	2	0.4	--	2 (100)	--	5:9
Total	524	100	87	398	39	

F-F: Clusters consisting of only females, M-F: Clusters consisting of both male and female individuals, M-M: Clusters consisting of only males, M:F ratio: Ratio of the number of male to female individuals.

**Table 4:** Characteristics of clusters of size 5 and above.

Cluster ID	Size	Age, Median (IQR)	Gender (M:F)	ART (Naïve: on ART)	Location (%)	Subtype (%)
C290	7	41.0 (26.0–50.0)	3:4	4:3	Central (14.3)	D (100)
					East (28.6)	
					GPC (42.9)	
					Non-GPC (14.3)	
C93	7	40.0 (36.0–44.0)	2:5	2:5	GPC (100)	A1 (100)
C272	6	22.5 (21.3–30.5)	4:2	6:0	GPC (83.3)	ISR (83.3)
					Non-GPC (16.7)	D (16.7)
C97	6	38.0 (35.5–40.5)	4:2	1:5	Central (16.7)	A1 (100)
					GPC (66.7)	
					Non-GPC (16.7)	
C448	5	43.0 (40.0–46.0)	2:3	0:5	GPC (100)	D (100)
C287	5	44.0 (30.0–55.0)	1:4	2:3	GPC (80)	D (100)
					Non-GPC (20)	
C281	5	29.0 (25.0–36.0)	3:2	2:3	GPC (20)	D (100)
					Non-GPC (80)	
C70	5	22.0 (19.0–26.0)	2:3	5:0	GPC (40)	A1 (100)
					Non-GPC (60)	

ISR, HIV-1 inter-subtype recombinants.

**Table 5:** Factors associated with the probability of belonging to a transmission cluster.

Covariate	Unadjusted OR (95% CI)	*p*-Value	Adjusted OR (95% CI)	*p*-Value
**Study location**				
Central	1			
East	0.86 (0.54–1.38)	0.5410	1.29 (0.66–2.40)	0.4415
GPC	3.59 (2.94–4.39)	<0.001	6.90 (5.22–9.21)	<0.001
Non-GPC	3.10 (2.53–3.81)	<0.001	5.12 (3.86–6.85)	<0.001
**Gender**				
Male	1			
Female	1.21 (0.89–1.17)	0.7720	1.28 (1.06–1.54)	0.0102
**Age**				
15–24	1			
25–34	1.01 (0.84–1.23)	0.8920	1.52 (1.16–2.0)	0.0024
35–44	0.97 (0.80–1.18)	0.7950	1.47 (1.12–1.93)	0.0050
45–54	1.04 (0.82–1.33)	0.7260	2.22 (1.61–3.08)	<0.001
>54	0.99 (0.75–1.30)	0.9460	1.60 (1.11–2.31)	0.0111
**ART**				
Naïve	1			
On ART	0.04 (0.03–0.05)	<0.001	0.025 (0.020–0.032)	<0.001
**Subtype**				
A1	1			
C	2.89 (2.31–3.62)	**<0.001**	0.91 (0.65–1.26)	0.5638
D	1.09 (0.93–1.28)	0.2890	1.18 (0.96–1.45)	0.1246
G	--	--	--	--
Intersubtype recombinants and other unknown HIV variants	0.97 (0.78–1.22)	0.8260	0.68 (0.49–0.92)	0.0139

CI; confidence interval.

## Supplementary Materials

The following are available online at https://www.mdpi.com/1999-4915/12/11/1283/s1, Table S1: Gender ratios shown at higher resolution for different cluster sizes.
