# Supplementary material for: The Molecular Epidemiology and Transmission Dynamics of HIV Type 1 in a General Population Cohort in Uganda
Source: Viruses. 2020 Nov 10;12(11):1283. doi: 10.3390/v12111283 (PMC7697205; doi:10.3390/v12111283)
Supplement: Supplementary file 1 [file viruses-12-01283-s001.pdf]

Table S1. Gender ratios shown at higher resolution for different cluster sizes

Cluster size	M:F ratio	Number of clusters
7	2:3	1
	2:5	1
6	4:2	2
	4:2	1
5	2:3	1
	1:4	1
	3:2	1
	2:3	1
4	1:3	5
	2:2	7
	3:1	1
3	1:0*	1
	0:3*	3
	1:2	46
	2:1	44
2	0:2*	84
	1:1	287
	1:0*	38

M:F, male: female; * pairs or clusters that consists of only males (M-M, male-male) or females (F-F, female-female)
